# CD38: an ecto-enzyme with functional diversity in T cells

**DOI:** 10.3389/fimmu.2023.1146791

**Published:** 2023-04-27

**Authors:** Alip Ghosh, Arshi Khanam, Krishanu Ray, Poonam Mathur, Ananya Subramanian, Bhawna Poonia, Shyam Kottilil

**Affiliations:** ^1^ Division of Clinical Care and Research, Institute of Human Virology, University of Maryland School of Medicine, Baltimore, MD, United States; ^2^ Division of Vaccine Research, Institute of Human Virology, University of Maryland School of Medicine, Baltimore, MD, United States; ^3^ Department of Environmental Science, Policy, and Management, University of California, Berkeley, Berkeley, CA, United States

**Keywords:** CD38, NAD+, T cells metabolism, HIV, mitochondrial function

## Abstract

CD38, a nicotinamide adenine dinucleotide (NAD)+ glycohydrolase, is considered an activation marker of T lymphocytes in humans that is highly expressed during certain chronic viral infections. T cells constitute a heterogeneous population; however, the expression and function of CD38 has been poorly defined in distinct T cell compartments. We investigated the expression and function of CD38 in naïve and effector T cell subsets in the peripheral blood mononuclear cells (PBMCs) from healthy donors and people with HIV (PWH) using flow cytometry. Further, we examined the impact of CD38 expression on intracellular NAD+ levels, mitochondrial function, and intracellular cytokine production in response to virus-specific peptide stimulation (HIV Group specific antigen; Gag). Naïve T cells from healthy donors showed remarkably higher levels of CD38 expression than those of effector cells with concomitant reduced intracellular NAD+ levels, decreased mitochondrial membrane potential and lower metabolic activity. Blockade of CD38 by a small molecule inhibitor, 78c, increased metabolic function, mitochondrial mass and mitochondrial membrane potential in the naïve T lymphocytes. PWH exhibited similar frequencies of CD38+ cells in the T cell subsets. However, CD38 expression increased on Gag-specific IFN-γ and TNF-α producing cell compartments among effector T cells. 78c treatment resulted in reduced cytokine production, indicating its distinct expression and functional profile in different T cell subsets. In summary, in naïve cells high CD38 expression reflects lower metabolic activity, while in effector cells it preferentially contributes to immunopathogenesis by increasing inflammatory cytokine production. Thus, CD38 may be considered as a therapeutic target in chronic viral infections to reduce ongoing immune activation.

## Introduction

Since the identification of CD38 as a thymocyte marker (1), this cell surface molecule has received significant attention as a T cell activation marker. A plethora of studies found higher expression of CD38 in various pathological conditions including cancers, autoimmune diseases and chronic viral infections, and identified its involvement in various biological processes including cell differentiation, cell migration, cytokine secretion, and apoptosis (2–6), suggesting it to be an important therapeutic target for treating cancers and autoimmune diseases (7). Monoclonal antibodies such as Daratumumab and Isatuximab have indeed shown encouraging results for the treatment of lymphoid cancers and autoimmune diseases (8–11). However, monoclonal antibody treatment, such as Daratumumab, results in clearance of CD38+ pathological cells by antibody dependent cellular cytotoxicity (ADCC), but it also increases risk of infections due to the depletion of CD38+ non-pathological immune cells (12). CD38 inhibitor such as 78c that functionally reduce the enzymatic activity of CD38 avoiding ADCC are now being tested for clinical interventions (13). Nevertheless, therapeutic potential of targeting CD38 in chronic infections such as HIV, HBV and HCV remains unknown.

With age, gradual increases in CD38 expression are linked to decline in NAD+ levels and mitochondrial dysfunction in various tissues in mice (14). Senescence-associated secretory phenotype (SASP) secreted by senescent cells during aging induce macrophages to proliferate and express CD38 leading to reduction of tissue NAD+ level (15). Conversely, inhibition of CD38 ameliorates this age-related metabolic dysfunction (13). Recently, association of CD38 upregulation with accelerated aging of immune cells has been reported in viral infections (16, 17), which might be associated with defective antiviral responses. Moreover, despite viral control with therapy, patients with chronic viral infections such as HBV and HIV carry a higher risk of developing inflammation-associated end-stage diseases, including cancer; therefore, an immunomodulatory therapy targeting CD38 to control excessive inflammation mediated by vigorous immune activation may demonstrate considerable therapeutic potential. Also, there is an advantage in targeting CD38 in chronic viral infections because it may have dual effect: reduce inflammation and enhance metabolic activity and restore function of effector T cells. However, since immunotherapies carry a risk of developing unprecedented complications, such as autoimmune diseases, it is important to decipher the precise nature and function of CD38 among various immune cell populations. This, in turn, will allow for identifying the advantages and disadvantages of targeting CD38 for therapeutic interventions.

Although CD38 has been demonstrated as a T cell activation marker in HCV and HIV infection (18–20), its function in different T cell subsets remain unclear. Some studies indicate CD38 is expressed in less than 10% of cytotoxic T lymphocytes (CTL) in PBMCs of healthy donors (HD) (20, 21), while others reported high expression on these cells (22–24). Dianzani et al. documented significantly higher expression of CD38 on CD45RA+ naive helper T cells and CTLs (24), indicating differential expression of CD38 on different cell types. Therefore, in this study we explored CD38 expression and its function in various T cell subsets and evaluated if CD38 is associated with cellular NAD+ metabolism, mitochondrial function and inflammatory cytokine production. Further, we aimed to understand the impact of targeting CD38 as a therapeutic option in patients with chronic viral infections, which might help in designing new therapeutic strategies.

## Methods

### Participants

Peripheral blood samples were acquired from patients and healthy donors after written informed consent. Samples from PWH were taken from the CHROME study (Protocol Number: HP-000813000; NCT0382391), approved by the University of Maryland, Baltimore Institutional Review Board. The pertinent inclusion criteria are men and women 18 years and older, CD4 count≥150/mm^3^, virally suppressed with ART (VL ≤ 50/mL). All healthy donors were between 18-50 years of age, tested negative for HIV, HBV or HCV infections, and presented without clinically relevant symptoms of any existing pathology at the time of sample collection. Peripheral blood mononuclear cells (PBMCs) were isolated using Ficoll-Paque (Sigma-Aldrich) density gradient centrifugation. Cells were subsequently resuspended in 90% fetal bovine serum (FBS, Gibco) plus 10% DMSO and stored in the vapor phase of liquid nitrogen until further use.

### Antibody staining and Flow cytometry

For all experiments, frozen PBMCs were thawed and rested for 16-18h in complete RPMI 1640 media at 37°C in a 5% CO_2_ incubator before starting the experiments. Phenotypic analyses of PBMCs were performed using surface antibody staining for 30 minutes at 4°C, using combinations of the following anti-human monoclonal antibodies: αCD3-PE/Dazzle594 (OKT3), αCD3-FITC (OKT3), αCD38-PE/Cy7 (HB-7), αCD19-BV650 (HIB19), αCD56-BV750 (5.1H11), αCD4-PerCP/Cy5.5 (OKT4), αCD8-AF700 (SK1), αCCR7-APC/Fire (G043H7), αCD45RO-BV711 (UCHL1), all purchased from BioLegend. After staining the cells with antibodies for the phenotypic markers cells were fixed in 1% paraformaldehyde and acquired in a flow cytometer. The functional capacity of T cells was determined by analyzing the intracellular cytokine production upon stimulation with overlapping HIV-Gag polyprotein peptides using αIFNγ-APC (4S.B3) and αTNFα-PE (MAb11) antibodies from BioLegend. After staining the cells for surface markers, cells were permeabilized with Cytofix/Cytoperm (BD Biosciences) followed by intra cellular cytokine staining and finally fixed in 1% paraformaldehyde. Live/dead cell discrimination was performed using Zombie Aqua fixable viability Kit (BioLegend). Data were acquired and compensated using Cytek Aurora (Cytek Biosciences) flow cytometer and further analyzed with FlowJo (version 10.7.1).

### Intracellular NAD+ level

Intracellular NAD+ level in T cells were measured using a chemiluminescence-based kit (NAD/NADH-Glo™ Assays; Promega, USA) following the manufacturer’s instructions. First, naïve and effector T cells were sorted from 10^7^ PBMCs collected from 7 healthy donors by flow cytometry cell sorter (BD Aria-III; BD Biosciences) using αCD3-APC (OKT3), αCCR7-APC/Fire (G043H7), αCD45RO-BV711 (UCHL1) antibodies purchased from BioLegend. Sorted cells were counted and 500,000 cells (in duplicate) were lysed according to the kit’s instructions to determine the mean intracellular NAD+ and NADH levels. Data was normalized by protein concentration determined by BCA protein estimation assay kit (Thermo) using a fraction of the same whole cell lysate that was used for NAD+/NADH measurement as recommended in the kit’s instruction.

The data was represented as NAD+ or NADH content per microgram of protein. For NAD+ measurement in CD4+ and CD8+ T cells after 24h treatment with 5μM 78c, cells were sorted by negative selection using magnetic bead-based isolation kits (EasySep Human CD4+/CD8+ T cell isolation kit, STEMCELL Technologies). Data were analyzed using GraphPad Prism software.

### Two-photon excitation fluorescence (2PEF) microscopy

Cells were stained with the following anti-human monoclonal antibodies; αCD38- PerCP/Cy5.5 (HB-7), αCD4-FITC (OKT4), αCD8-FITC (RPA-T8) from BioLegend. Two-photon excitation fluorescence imaging was performed in a scanning microscope (ISS Q2 with Galvano-scanner unit coupled to an Olympus IX70). The two-photon excitation was generated by a femtosecond laser (Calmar Laser Inc., 780nm, pulse-width 90fs, repetition rate of 50MHz). A computer-controlled ISS intensity modulation unit (based on Glan-Thompson polarizer and half-wave plate) was used to precisely regulate the excitation power from the femtosecond laser. The excitation light was reflected by a dichroic mirror to a high-numerical-aperture (NA) water objective (60x; NA 1.2) and focused onto the cells attached on a cover glass slide. The fluorescence was collected by avalanche photodiodes through a 750nm short-pass filter, dichroic beam splitters and band-pass filters (Chroma) for recording the fluorescence in the spectral regions of interest in three separate detection channels (435 – 485 nm for NADH; 502 – 548nm for FITC; and 650 – 720nm for PerCP-Cy5.5). The data acquisition was enabled by a B&H SPC-150 card operated in a photon time-tag time-resolved (TTTR) mode. ISS VistaVision software for data acquisition and data processing was used for fluorescence imaging experiments by two-photon excitation. Average intensity per cell (measured by photon counts) from the CD38-PerCP-Cy5.5 and NADH channels were presented in bar diagrams from 3 different sections of the naïve and effector CD4+ and CD8+ T cell subsets.

### Measurement of metabolic activity of T cells by extracellular flux analyzer

PBMCs were thawed and rested for 16-18h in complete RPMI 1640 media at 37°C in a 5% CO_2_ incubator. CD3+ T cells from 6 HDs were separated using, EasySep Human T cell isolation kit (STEMCELL Technologies). Then, naïve and effector T cells were sorted by flow cytometry using αCCR7-FITC (G043H7), αCD45RO-APC/Cy7 (UCHL1) antibodies from BioLegend. Oxygen consumption rate (OCR) and extracellular acidification rate (ECAR) were measured in a Mitostress test conducted in a Seahorse XFe96 extracellular flux analyzer (Agilent) following the standard protocol from the manufacturer. Briefly, 5x10^5^ cells (at least 3 replicates) were seeded in a PolyD-Lysine coated plate (Agilent). Cells were initially incubated in XF RPMI medium supplemented with glutamine (2mM), glucose (10mM) and pyruvate (1mM) (all reagent from Agilent). Cells were then treated sequentially with Oligomycin (1 μM) to block ATP production, followed by the uncoupling agent FCCP (fluoro-carbonyl cyanide phenylhydrazone, 1.5 μM), to dissipate proton gradients and allow electron transport and oxygen consumption to operate at maximal rate. This elevated OCR was suppressed by Rotenone/Antimycin (0.5 μM), showing that respiration was mitochondrial. Basal OCR and ECAR measurement were taken before addition of Oligomycin, maximal mitochondrial respiration was measured after addition of FCCP and mitochondrial spare capacity was measured by subtracting basal respiration from maximal respiration.

### Analysis of mitochondrial mass, mitochondrial superoxide and mitochondrial membrane depolarization

After thawing frozen PBMCs and resting for 16-18hr cells were treated with or without 5µM 78c for 24h. Cells were then stained for live/dead discrimination followed by staining for phenotypic surface markers (as described above) at 37°C in a 5% CO_2_ incubator for 20 minutes. At the end of surface staining, prewarmed mitochondria-specific dyes were added and incubated for 10 minutes. Finally, cells were washed, acquired immediately in Cytek Aurora flow cytometer. Data were acquired using Cytek Aurora and further analyzed with FlowJo (version 10.7.1). Mitochondrial mass was determined by measuring the mean fluorescence intensity (MFI) of mitochondria-specific dye Mitotracker Green (MTG; 100µM) among the naïve and effector subsets of CD4+ and CD8+ T cells. Frequency of mitochondrial superoxide positive cells were determined by staining the lymphocytes with MitoSox Red dye (100µM), which specifically detects superoxide content. Mitochondrial depolarization was determined by the red to green ratio of MFIs of mitochondrial membrane potential dependent dye JC1 (1µg/ml) among the naïve and effector subsets of CD4+ and CD8+ T cells. All the mitochondria specific dyes were purchased from Thermo.

### HIV Gag-specific T cell response

PBMCs from 8 virally suppressed PWH individuals were used to measure antigen-specific cytokine production. PBMCs were cultured at 37°C in a 5% CO2 incubator for 5 days in complete RPMI-1640 medium at a final concentration of 1 × 10^6^ cells/ml in the absence or presence of overlapping peptides (1 μg/ml) of HIV-Gag polyprotein (PepMix™ HIV-1 (GAG) Ultra) from JPT Peptide Technologies GmbH. The peptide mix contains 150 overlapping peptides (15mers with 11 aa overlap) through Gag polyprotein of HIV. PBMCs were re-stimulated with overlapping peptides on day 4 and Brefeldin A and Monensin (BD Biosciences) were added after 1 hour of re-stimulation, and the cell cultures were continued for an additional 16 hours. Cells incubated without Gag peptides but with DMSO of equivalent concentration as used to dissolve the peptides served as negative controls. Recombinant IL-2 (20 IU/ml, Tecin, Biological Resources Branch, NIH) was added to Gag-stimulated or control cells at day 1 of culture. To inhibit CD38 functionally, 5μM of a small molecule inhibitor of CD38, 78c (Millipore, Sigma) was used. Cells were stained with surface markers and intracellular cytokines for flow cytometry and data was acquired and analyzed as described above.

### Statistical analysis

Data were analyzed on GraphPad Prism (V9.2.0). Statistical significance was tested by Kruskal-Wallis test (un-paired nonparametric data) or Friedman test (paired and nonparametric data) with *post hoc* analysis (Dunn’s multiple comparison test) for data with multiple groups. For comparison of the mean between two groups, Mann-Whitney test was used for nonparametric data sets. Paired t-test was used to verify the statistical significance for paired samples.

## Results

### CD38 expression varies on different subsets of lymphocytes among healthy donors

**Figure 1:**
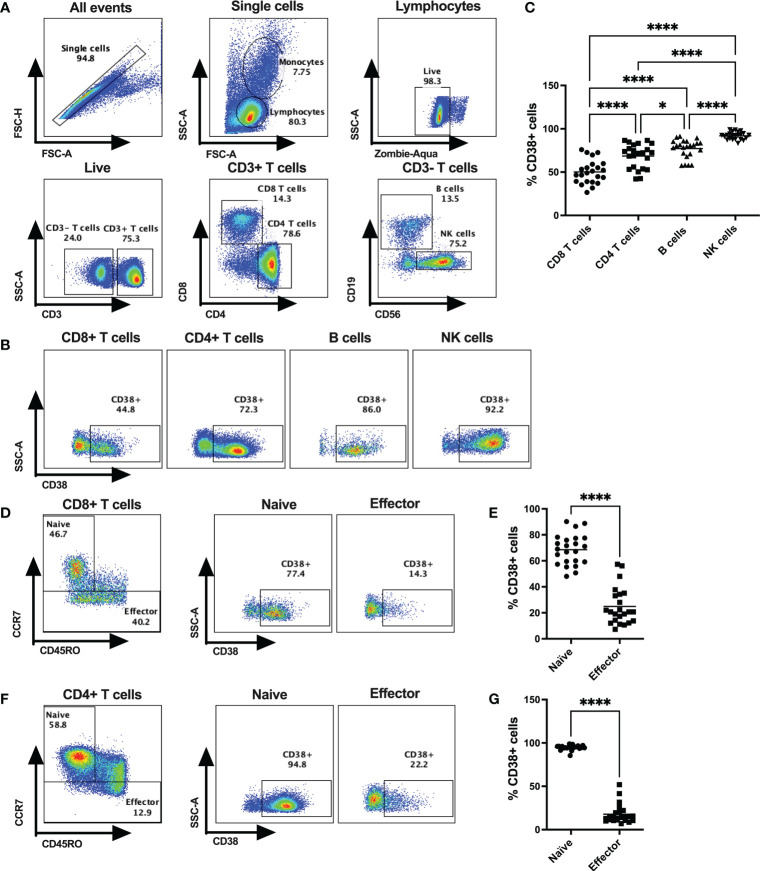
CD38 expression on various subsets of cytotoxic T lymphocytes in HDs. **(A)** Gating strategies for CD4+ T cells, CD8+ T cells, B cells and NK cells. **(B)** Representative dot plots for % CD38+ cells among CD4+ T cells, CD8+ T cells, B cells and NK cells in PBMCs of a HD. **(C)** Frequencies of CD38+ cells among CD4+ T cells, CD8+ T cells, B cells and NK cells in PBMC of HDs (n=23). **(D)** Gating strategy for Naïve and Effector subsets based on surface expression of CD45RO and CCR7 of CD8+ T cells and representative dot plots for %CD38+ cells among them. **(E)** Proportion of CD38+ cells among Naïve and Effector subsets of CD8+ T cells in PBMC of HDs. **(F)** Gating strategy for Naïve and Effector subsets based on surface expression of CD45RO and CCR7 of CD4+ T cells and representative dot plots for %CD38+ cells among them. **(G)** Proportion of CD38+ cells I among Naïve and Effector subsets of CD4+ T cells in PBMC of HDs. Horizontal black lines in dot plots represent mean values. In **(C)**, Kruskal-Wallis test was performed for statistical analysis with *post hoc* test (Dunn’s multiple comparison test). Adjusted P values; * <0.05, **** <0.0001. In **(E, G)** Wilcoxon matched-pairs signed rank test were performed. P values; **** <0.0001.

Frequencies of CD38+ cells were verified on lymphocytes (CD4+ T cells, CD8+ T cells, B cells, NK cells) of 23 healthy donors (HD; 17 male and 6 female) by flow cytometry (**Figure 1**). All four types of lymphocytes express CD38, but the extent of CD38 expression varied substantially among the immune cell subtypes. The lowest frequency of CD38+ cells was observed on CD8+ T cells (mean %= 52.36 ± 14.94) followed by CD4+ T cells (mean %= 70.54 ± 13.38), B cells (mean %= 75.57 ± 10.36), and NK cells (mean %= 91.09 ± 3.98) (**Figures 1A–C**). Next, we investigated the expression of CD38 among the naïve and effector T cell subsets based on CD45RO and CCR7 expression. Intriguingly, our data showed that CCR7+CD45RO- CD8+ T cells, that are primarily comprised of naïve cells, had the highest CD38 expression (mean %= 68.58 ± 11.74) compared to CCR7-CD45RO+/- effector cells that are primarily comprised of effector memory and terminally differentiated effector cells (mean %= 24.92 ± 14.24) (**Figures 1D, E**). Similar distribution of CD38 expression were also observed in CD4+ T cell subsets where majority of the naïve cells had higher CD38 expression (mean %= 94.86 ± 2.82) compared to effector cells (mean %= 17.78 ± 10.9) (**Figures 1F, G**). Since a considerable proportion of naïve/resting T cells (presumably inactive) had high expression of CD38, it may not solely be an activation marker and instead have diverse functions in different cell types.

### CD38 modulates intracellular NAD+ levels in T lymphocytes

**Figure 2:**
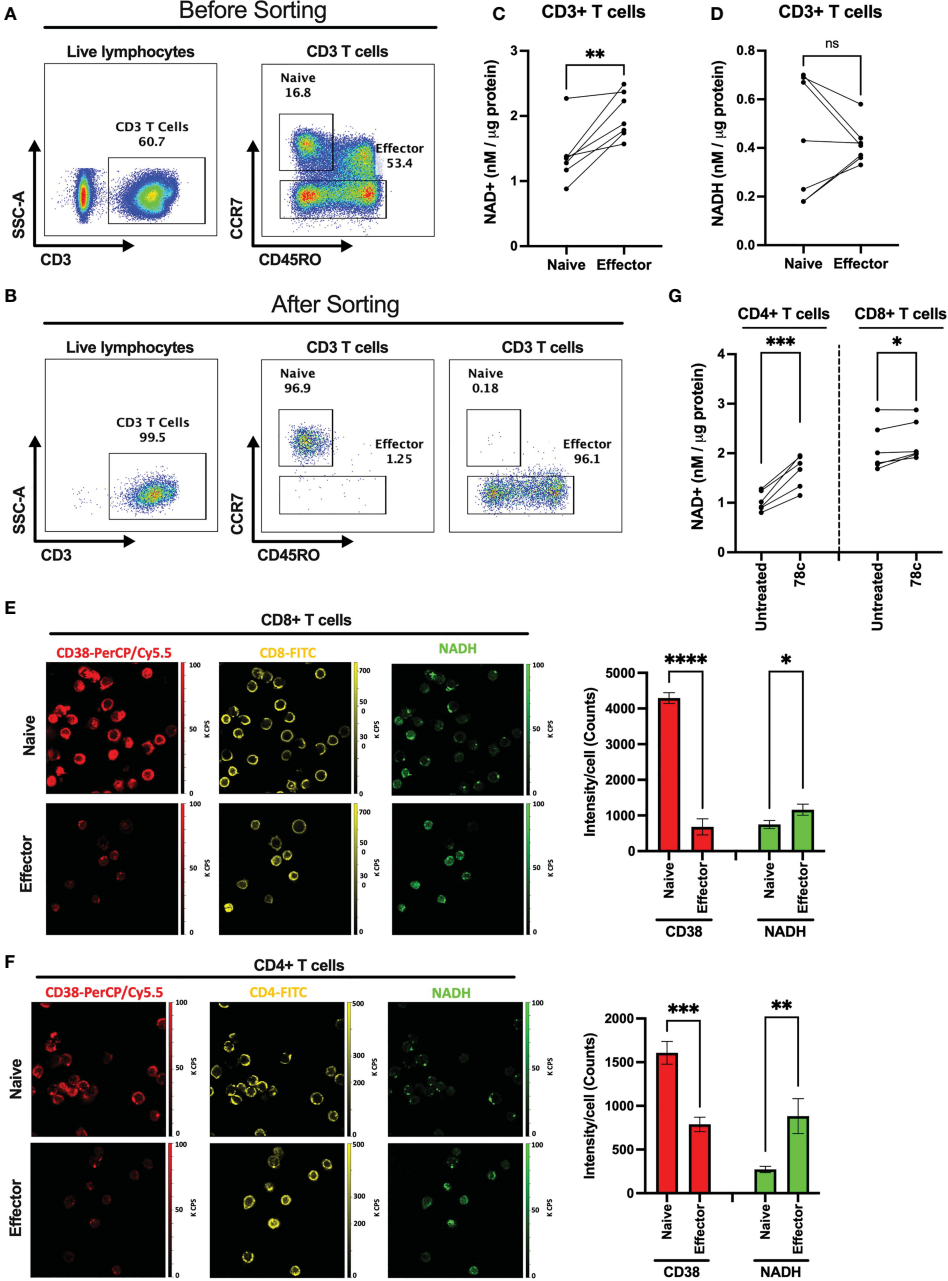
Comparison of intracellular NAD+ and NADH levels between naïve and effector T cell subsets. Gating strategies for naïve (CCR7+CD45RO-) and effector (CCR7-CD45RO+/-) CD3+ T cell subsets before **(A)** and after **(B)** cell sorting. **(C)** Intracellular NAD+ level in naïve and effector T cell subsets. **(D)** Intracellular NADH level in naïve and effector T cell subsets. 2PEF images of CD8+ **(E)** and CD4+ **(F)** (yellow) cells expressing CD38 (red) and intracellular NADH (green) among naïve and effector subsets sorted by flow cytometry as indicated above. Bar diagrams represent average of 3 different sections from each of naïve and effector T cell subsets and error bars represent standard deviation. Image sizes were 75µm x 75µm. **(G)** Intracellular NAD+ level in CD4+ and CD8+ T cells after treating them with 5μM CD38 inhibitor, 78c, for 24h. Paired t-test was performed for statistical analysis in C, D and **(G)** Unpaired t-test was performed for statistical analysis in E and **(F)** P values; * <0.05, ** <0.01, *** <0.001, **** <0.0001. ns – not significant.

Since one of the major functions of CD38 is hydrolysis of NAD+, we hypothesized that overexpression of CD38 may differentially modulate intracellular NAD+ level among naïve and effector T cells. We measured the intracellular NAD+ and NADH levels in purified CD3+ naïve and effector T lymphocytes (**Figures 2A–D**) in HDs (n=7). First, we sorted the naïve (CCR7+CD45RO-) and effector (CCR7-CD45RO+/-) CD3+ T cells based on CCR7 and CD45RO staining. Then, intracellular NAD+ and NADH levels were measured by a chemiluminescence-based assay. Interestingly, intracellular NAD+ levels were lower among the naïve T cells (Mean = 1.38 ± 0.43 nM/μg protein) compared to effector T cells (Mean = 2.01 ± 0.35 nM/μg protein) in all individuals and this difference was highly significant (p<0.005) (**Figure 2C**). However, differences in NADH levels were not significant between naïve (Mean = 0.44 ± 0.25 nM/μg protein) and effector (Mean = 0.42 ± 0.08 nM/μg protein) T cells (**Figure 2D**). We further evaluated intracellular NADH levels in sorted naïve and effector cells qualitatively by two-photon excitation fluorescence imaging microscopy (2PEF). Again, naïve CD8+ and CD4+ T cells had substantially higher CD38 expression and lower NADH expression compared to their effector counterparts (**Figures 2E, F**). However, despite notable changes in NAD+ and NADH levels, no significant difference was observed in the NAD+/NADH ratio between the naïve and effector T lymphocytes (**Supplementary Figure S1**). We previously showed lower intracellular NAD+ levels in CD38+ compared with CD38- T cells (19) indicating CD38 protein expression on T lymphocyte possibly modulates intracellular NAD+ levels. Here we demonstrate that 24h of treatment with a small molecule functional inhibitor of CD38, 78c, resulted in significant increase in intracellular NAD+ levels in CD4+ and CD8+ T cells (**Figure 2G**). Interestingly, the increase in NAD+ levels due to 78c treatment were more in CD4+ T cells compared to CD8+ T cells. Perhaps, this was because CD4+ T cells had higher CD38 expression than CD8+ T cells as shown in **Figure 1**. It would be interesting to measure the change in NAD+ levels with 78c treatment on naïve and effector subsets of CD4+ and CD8+ T cell separately, but we could not verify it due to inadequate cell number in the sorted effector T cell subsets. Nonetheless, these data confirms a direct role of CD38 in depleting NAD+ levels in T cells.

### CD38 modulates metabolic activity of naïve T lymphocytes

**Figure 3:**
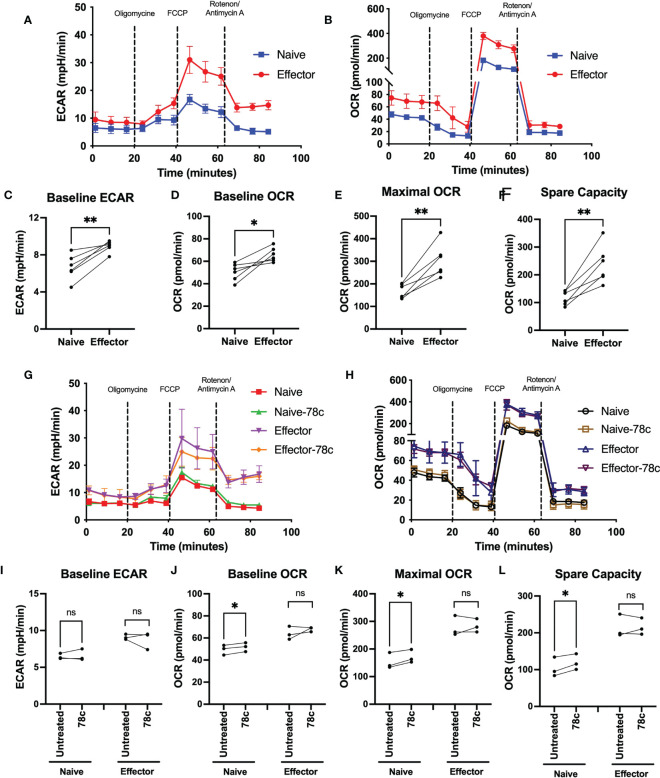
Assessment of metabolic activity of T lymphocyte subsets in HDs. Representative figure of **(A)** ECAR and **(B)** OCR among naïve and effector subsets of CD3+ T cells. Plots for **(C)** baseline ECAR, **(D)** baseline OCR, **(E)** maximal OCR and **(F)** spare capacity in paired naïve and effector T cells among HDs. In each plot, dots represent individual samples and connected lines represent paired values in naïve and effector cells. Representative figure of **(G)** ECAR and **(H)** OCR among naïve and effector subsets of CD3+ T cells treated with or without 78c for 24h. Plots for **(I)** baseline ECAR, **(J)** baseline OCR, **(K)** maximal OCR and **(L)** spare capacity in paired naïve and effector T cells among HDs treated with or without 78c for 24h. In each plot, dots represent individual samples and connected lines represent paired values in naïve and effector cells. Paired t-test was performed for statistical analysis. P values; * <0.05, ** <0.01, ns – not significant. HD- Healthy donor, ECAR - extracellular acidification rate, OCR- oxygen consumption rate.

Since our data indicated that NAD+ levels vary significantly among naïve and effector T lymphocytes, we examined the influence of CD38 on OXPHOS and glycolysis among T cell subsets of HDs by measuring oxygen consumption rate (OCR) and extracellular acidification rate (ECAR) using the Seahorse extracellular flux analyzer. OCR and ECAR indicate the activity of OXPHOS and glycolysis, respectively. As indicated in **Figure 3** the naïve subset of T lymphocytes had significantly lower basal ECAR (**Figures 3A, C**) and OCR (**Figures 3B, D**) compared to their corresponding effector T lymphocytes. Moreover, the maximal mitochondrial respiration induced by uncoupler FCCP treatment was much higher in the effector T cells compared to naïve cells (**Figure 3E**). In addition, the spare capacity of the effector T cells, which measures the potential of a cell to produce ATP immediately upon demand, was substantially higher than naïve T cells (**Figure 3F**). Furthermore, CD38 inhibition by 78c treatment for 24h increased baseline OCR (**Figures 3H, J**), maximal respiration (**Figures 3H, K**) and spare capacity (**Figures 3H, L**) in naïve T cell but not in effector T cells. However, CD38 inhibition did not have any significant effect on baseline ECAR (**Figures 3G, I**) on either subset of T cells. These data suggest that at baseline, effector cells are more metabolically active than naïve T cells and are better equipped to produce ATP rapidly by utilizing both oxidative phosphorylation and glycolysis.

### CD38 modulates mitochondrial function of T lymphocytes

**Figure 4:**
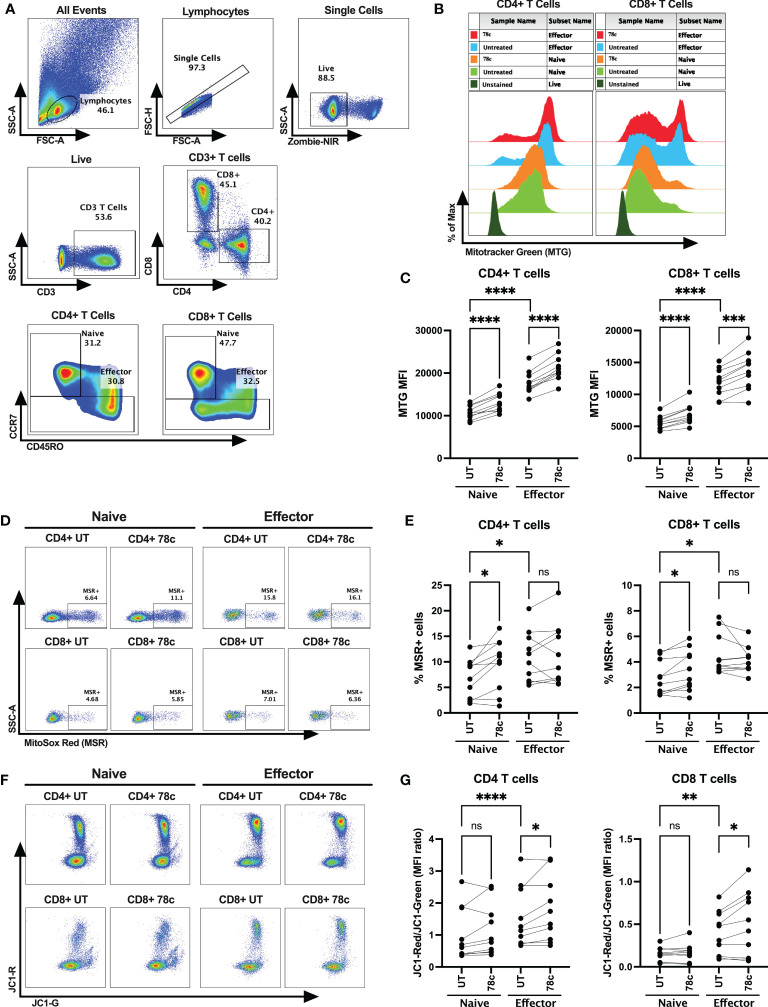
Assessment of mitochondrial functions using mitochondria-specific dyes Mitotracker Green (MTG), Mitosox Red (MSR) and JC-1 among the T cell subsets of HDs. **(A)** Gating strategy for identifying naïve and effector CD4+ and CD8+ T cells **(B)** Representative overlay histogram plot to assess MFI of MTG among naïve and effector subsets of CD4+ and CD8+ T cells in the presence and absence of 78c treatment. **(C)** Comparison of MTG-MFI among naïve and effector subsets of CD4+ and CD8+ T cells in the presence or absence of 78c treatment. **(D)** Representative dot plots for %MSR+ cells among naïve and effector subsets of CD4+ and CD8+ T cells in the presence or absence of 78c treatment. **(E)** Comparison of % MSR+ cells among naïve and effector subsets of CD4+ and CD8+ T cells in the presence or absence of 78c treatment. **(F)** Representative dot plots for JC1-Red (aggregates)/JC1-Green (monomers) staining among naïve and effector subsets of CD4+ and CD8+ T cells in the presence or absence of 78c treatment. **(G)** Comparison of the MFI ratio of JC1-Red/JC1-Green fluorescence among naïve and effector subsets of CD4+ and CD8+ T cells in the presence or absence of 78c treatment. In each plot, dots represent individual samples and lines represent paired values. Paired t-test was performed for statistical analysis. P values; * <0.05, ** <0.01, *** <0.001, **** <0.0001, ns – not significant.

Next, we determined mitochondrial functions among the T cell subsets using mitochondria-specific dyes Mitotracker Green (MTG), Mitosox Red (MSR) and JC-1 among HDs. MTG stains all mitochondria inside the cells by selectively diffusing into mitochondria, irrespective of mitochondrial membrane potential, giving an estimate of total mitochondrial mass. Oxidation of MSR by mitochondrial superoxide (mitochondria is the major source of superoxide generated by the electron transport chain (ETC)) produces red fluorescence, giving the estimate of mitochondrial superoxide content. In highly polarized mitochondria, JC1 aggregates and produces red fluorescence, whereas in depolarized mitochondria it remains in monomer form and emits green fluorescence. Thus, depolarized cells with lower mitochondrial membrane potential produce higher green fluorescence, leading to a lower JC1-red to JC1-green ratio. Mitochondrial mass, determined by the MFI values of MTG, was significantly lower among the naïve cells compared to effector CD4+ (p<0.0001) and CD8+ T cells (p<0.0001) (**Figures 4A–C**). In parallel, lower percentage of superoxide positive cells were observed among naïve cells compared to effectors in CD4+ T cells (p<0.05) and CD8+ T cells (p<0.05) (**Figures 4A, D, E**). Interestingly, cells with high mitochondrial mass were found to be MSR+ cells in both naïve and effector T cell subsets (**Supplementary Figures S2A–C**). Surprisingly, the MFI ratios of JC1-red/JC1-green were substantially lower among the naïve cells compared to effector cells in both CD4+ (p<0.0001) and CD8+ T cell subsets (p<0.01) (**Figures 4A, F, G**). Moreover, 24h treatment of 78c, resulted in remarkably increased mitochondrial mass among all T cell subsets in both CD4+ and CD8+ (p<0.0001) T cells (**Figures 4A–C**). However, 78c treatment increased mitochondrial superoxide only in the naïve (p<0.001) subsets but not in effector cells of both CD4+ and CD8+ T cells (**Figures 4A, D, E**). Furthermore, mitochondrial membrane potential improved marginally in effector subsets of both CD4+ and CD8+ (p<0.05) T cells (**Figures 4A, F, G**), but not in naïve subsets. These data indicate that naïve T cells maintain a metabolically hypoactive pool of mitochondria while the effector cells maintain metabolically active and more polarized pool of mitochondria, and CD38 inhibitor can partially modulate mitochondrial functions within 24h of treatment.

### CD38 is required for functional activation and cytokine response by T cells upon viral peptide stimulation

**Figure 5:**
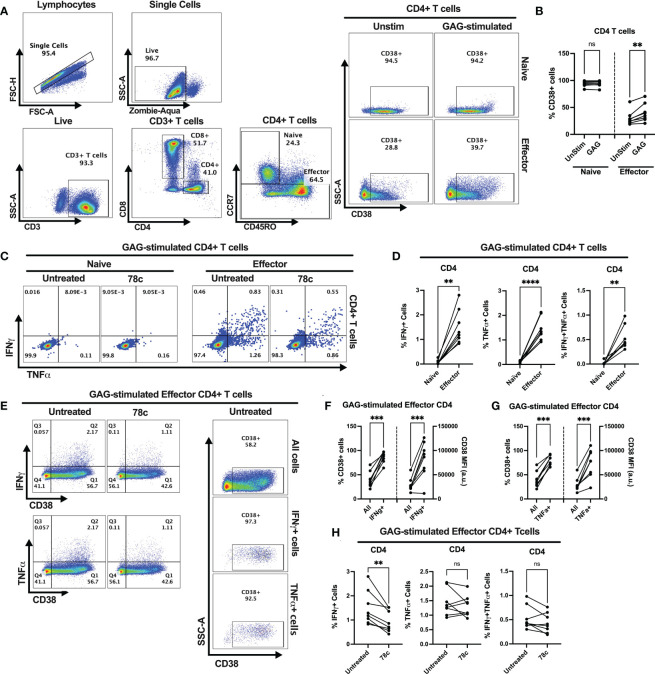
Intracellular cytokine production and change in CD38 expression in response to HIV Gag-specific peptide stimulation among the CD4+ T cell subsets of 8 PWH on ART. **(A)** Representative flow-plots for gating strategy to identify CD38+ cells among CD4+ T lymphocytes stimulated with Gag-peptide. **(B)** Percentages of CD38+ cells among naïve and effector CD4+ T cells with or without Gag-peptide stimulation. **(C)** Representative flow-plots for IFNγ+ and TNFα+ cells among the Gag-peptide stimulated naïve and effector CD4+ T cells in presence and absence of CD38 inhibitor, 78c. **(D)** Comparison of the percentages of IFNγ+, TNFα+ and IFNγ+TNFα+ cells between Gag-peptide stimulated naïve and effector CD4+ T cells. **(E)** Representative flow-plots for cytokine production on CD38+ cells in the presence or absence of 78c treatment and the % CD38+ cells among global (All cells), IFNγ and TNFα producing Gag-peptide stimulated effector CD4+ T cells. Comparison of CD38 expression (% and MFI) between global (All) vs. IFNγ+ **(F)** and global vs. TNFα+ **(G)** Gag-peptide stimulated effector CD4+ T cells, respectively. **(H)** Percentage of IFNγ+, TNFα+ and IFNγ+TNFα+ cells in the absence (untreated) and presence of 78c in Gag-peptide stimulated effector CD4+ T cells. In each plot, dots represent individual samples. Paired t-test (for parametric data) or Wilcoxon test (for nonparametric data) was performed for statistical analysis. P values; ** <0.01, *** <0.001, **** <0.0001, ns – not significant.

**Figure 6:**
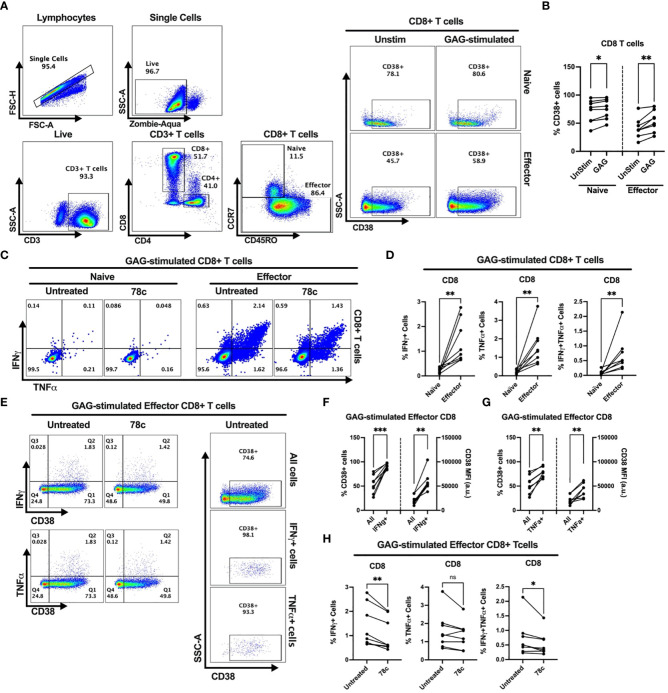
Intracellular cytokine production and change in CD38 expression in response to HIV Gag-specific peptide stimulation among the CD8+ T cell subsets of 8 PWH on ART. **(A)** Representative flow-plots for gating strategy to identify CD38+ cells among T lymphocytes stimulated with Gag-peptide. **(B)** Percentages of CD38+ cells among naïve and effector CD8+ T cells with or without Gag-peptide stimulation. **(C)** Representative flow-plots for IFNγ+ and TNFα+ cells among the Gag-peptide stimulated naïve and effector CD8+ T cells in presence and absence of CD38 inhibitor, 78c. **(D)** Comparison of the percentages of IFNγ+, TNFα+ and IFNγ+TNFα+ cells between Gag-peptide stimulated naïve and effector CD8+ T cells. **(E)** Representative flow-plots for cytokine production on CD38+ cells in the presence or absence of 78c treatment and the % CD38+ cells among global (All cells), IFNγ and TNFα producing Gag-peptide stimulated effector CD8+ T cells. Comparison of CD38 expression (% and MFI) between global (All) vs. IFNγ+ **(F)** and global vs. TNFα+ **(G)** Gag-peptide stimulated effector CD8+ T cells, respectively. **(H)** Percentage of IFNγ+, TNFα+ and IFNγ+TNFα+ cells in the absence (untreated) and presence of 78c in Gag-peptide stimulated effector CD8+ T cells. In each plot, dots represent individual samples. Paired t-test (for parametric data) or Wilcoxon test (for nonparametric data) was performed for statistical analysis. P values; * <0.05, ** <0.01, *** <0.001, ns, not significant.

Our data indicates that CD38 likely plays different role in naïve and effector cells. However, the lower expression of CD38 among the effector compared to naïve T cells raises the question of the role that CD38 plays in effector cells. Does the expression and function of CD38 change when the effector cells encounter microbial antigens? To investigate this, we performed intracellular cytokine production assays by stimulating PBMCs of PWH with HIV Gag-specific peptides in the presence or absence of CD38 inhibitor 78c. First, we compared the percentages of CD38+ cells between HDs and PWH among naïve and effector subsets of CD4+ and CD8+ T cells. There were no significant differences in the percentages of CD38+ cells between HDs and PWH among the naïve, and effector T cell subsets (**Supplementary Figures S3A, B**). Next, we compared the changes in CD38 expression on the naïve and effector subsets of CD4+ and CD8+ T cells after HIV Gag-peptide stimulation to that of unstimulated cells. No change in the percentage of CD38+ cells was observed in CD4+ naïve T cells after Gag-peptide stimulation (**Figures 5A, B**), while mild increase was observed in CD8+ (p<0.05) naïve T cells (**Figures 6A, B**). In contrast, substantial increases in the percentages of CD38+ cells were detected in both CD4+ (**Figures 5A, B**) and CD8+ effector T cell subsets (**Figures 6A, B**) upon Gag-peptide stimulation. As expected, the naïve CD4+ and CD8+ T cell subsets barely produced IFNγ or TNFα cytokines upon Gag-peptide stimulation (**Figures 5C, D** and **Figures 6C, D**). However, the percentages of IFNγ+, TNFα+ and IFNγ+TNFα+ cells were significantly higher among the effector CD4+ and CD8+ T cells compared to their naïve counterparts (p<0.01 in all cases) (**Figures 5C, D** and **Figures 6C, D**). Intriguingly, IFNγ and/or TNFα producing Gag-peptide stimulated effector cells (for both CD4+ and CD8+ T cell subsets) had remarkably higher CD38 expression (measured by both frequency and MFI of CD38+ cells, p<0.01 in all cases) compared to the global (All) effector T cell subsets (**Figures 5E–G** and **Figures 6E–G**). Moreover, in the presence of CD38-inhibitor, 78c, the percentages of IFNγ producing cells in Gag-peptide stimulated effector CD4+ T cells (**Figures 5C, H**) and IFNγ+ and IFNγ+TNFα+ cells in Gag-peptide stimulated effector CD8+ T cells (**Figures 6C, H**) were significantly declined compared to control. These data suggest that HIV gag induces CD38 expression on effector T cells, which in turn triggers a pro-inflammatory cytokine response in these cells, which may or may not be contributing to viral control.

## Discussion

Majority of the existing literature on CD38 describes it as an activation marker on T lymphocytes. Here we have shown that, during pathogenic stimulation CD38 expression increases on effector cells resulting in functional activation and cytokine production, but at physiological conditions CD38 is highly expressed among a vast majority of the resting/naïve T cell subset compared to the effector T cell subset where it plays different roles including modulation of intracellular NAD+ levels, mitochondrial function and metabolic activity of the cells. This study revealed, naive T cells that express highest levels of CD38 are comprised of depolarized mitochondria and are metabolically inactive compared to effector T cells which have lower CD38 expression and polarized mitochondria with high metabolic activity. In pathologic states, such as HIV infection, effector T cells express CD38 in response to viral protein stimulation, which leads to secretory dysregulation of T cells facilitating an antigen specific pro inflammatory cytokine response.

Our data indicated that at physiological condition, CD38 expression is significantly lower in CD4+ and CD8+ T cells compared to the B cell and NK cell compartments indicating a possible distinct functional role of CD38 in different subsets of lymphocytes. Interestingly, among T cell subsets, naïve T cells expressed high level of CD38 which is likely to regulate the metabolic function of the naïve cells by reducing their intracellular NAD+ levels. Intracellular NAD+ levels are crucial for the function of many NAD+ consuming enzymes, such as poly-ADP Ribose polymerases (PARPs), ADP-ribosyltransferases (ARTs) and SIRTUINs (7). In addition, NAD+ is a cofactor for multiple metabolic enzymes in anabolic and catabolic reactions (25). Our results indicate that intracellular NAD+ levels in T cells is modulated by CD38, as naïve T cells expressing higher CD38 protein had lower intracellular NAD+ and NADH levels compared to the effector cells with lower CD38 expression. However, we did not observe any significant association of CD38 expression with the NAD+/NADH ratio, which is a critical component of the redox state of the cells, metabolic activities, and overall health of the cells (26). Other molecular mechanisms associated to NAD+-biosynthesis or NAD+ salvage pathways might be responsible for maintaining a stable intracellular NAD+/NADH ratio. Our previously published result by analyzing the intracellular NAD+ level in CD38+ and CD38- T cell compartments further confirm that (19). As expected, intracellular NAD+ levels were much lower in CD38+ T cells compared to their CD38- T cell counterpart, revealing the involvement of CD38 in regulating intracellular NAD+ levels. Moreover, blockade of CD38 by its inhibitor, 78c, increased the intracellular NAD+ level within 24h, which further confirms CD38 dependent NAD+ regulation in T cells. Our findings are supported by a recent study which shows that 78c rescues the intracellular NAD+ level by specifically inhibiting the hydrolytic function of CD38 (13). Thus, CD38-mediated NAD+ decline in cells may partially alter cellular metabolic functions. Although we have established a direct impact of CD38 expression on intracellular NAD+ level, various other molecular pathways are involved in modulation of intracellular NAD+ level including other NAD+ consuming enzymes such as PARPs and SIRTUINs and key enzymes in NAD+ biosynthesis and NAD+ salvage pathways (7). Also, animal models have shown that CD38-/- mice could survive for a long time with relatively compromised immune functions (27–30), indicating CD38 is not the only determining factor for the level of intracellular NAD+ and the metabolic fate of the naïve T cells. We did not verify the impact of other key enzymes in this study which could also be responsible for the metabolic status of these cells.

T cell activation, proliferation and subsequent cytokine production warrant higher demands of bioenergy, which is primarily met by cellular metabolism through oxidative phosphorylation (OXPHOS) and glycolysis. Both these metabolic pathways utilize NAD+ and NADH as co-factors for electron transfer. Interestingly, our data indicate that the naïve T cells with higher CD38 expression and concomitant reduced intracellular NAD+ level were metabolically dormant with minimal utilization of OXPHOS and glycolysis. Unlike the resident cells of any other organs in the human body, a majority of circulating immune cells, especially the naïve T cells of adaptive immunity, remain functionally inactive until antigen recognition. This could explain why we saw low metabolic activity (i.e., low OCR and ECAR) in naïve T cells. In parallel to increased intracellular NAD+ level, CD38 inhibition also increased baseline oxidative phosphorylation, maximal respiration and mitochondrial spare capacity, particularly in naïve T cells. Although, the increase in these parameters were persistent in multiple samples, the changes were quantitatively low, indicating increase in intracellular NAD+ level by CD38 inhibition may only partially contribute to modulate the metabolic activity of the naïve T cell subset; other molecular mechanism may be involved. The ETC in OXPHOS maintains the proton gradient across the mitochondrial inner membrane establishing the mitochondrial membrane potential. ETC is also the site for mitochondrial superoxide production (31). Alteration of the level of these parameters often affects mitochondrial function in a cell. Mitochondrial function is a sensitive and dynamic cellular process that is easily influenced by various factors including stress due to freezing and thawing and *in vitro* culture conditions. To avoid these confounders, we performed all our experiments after resting the cells for 16-18h after thawing and using same culture conditions. Therefore, the differences in the mitochondrial mass, superoxide level and mitochondrial membrane potential observed between naïve and effector cells under the same culture condition indicate that distinct mitochondrial functions exist among different immune cell subsets, which is likely modulated by the cells’ CD38 expression. Naïve T cells apparently maintain a depolarized pool of mitochondria with relatively lower level of mitochondrial mass and mitochondrial superoxide, which is probably physiological, as almost all the samples included in the experiment had uniform data. Moreover, CD38 inhibitor led to increased mitochondrial mass, superoxide content and mitochondrial membrane potential which may explain the increase in oxidative phosphorylation in the naïve T cell subsets. Our data corroborate with the previous finding that naïve T cells maintain a low mitochondrial membrane potential before exposure to antigenic stimulation (32). In fact, Marek et al. had previously reported that naïve CD4+ T cells even maintain a reduced plasma membrane potential in comparison to effector cells (33). On the contrary, CD38 is expressed in low proportions among circulating effector T cells, which maintain relatively higher intra cellular NAD+ levels and higher metabolic activity with a pool of polarized mitochondria.

During pathological conditions such as in HIV infection, naïve T cells are activated by antigenic exposure, and differentiate into various types of effector cells including helper T cells, cytotoxic T cells, and regulatory T cells (34), producing cell-specific cytokines & chemokines. During persistent antigenic exposure, CD38 is upregulated and plays a crucial role in driving many of the biological processes in activated effector T cells. For instance, during the acute phase of HCV or HIV infection, virus-specific T cells express higher CD38 in conjunction with Ki67, a marker of cell proliferation, indicating CD38 may induce cell proliferation in T cells (35). It has been shown that HIV-specific IFNγ producing CD4+ T cells in PWH are predominantly CD38+ and Ki67+ (19, 36). Frequency of apoptotic CD8+ T cells is also higher in PWH with high CD38 expression compared to those with low CD38 expression (37). The enigmatic expression of CD38 on both inactive naïve and activated effector cells emphasize that CD38 may have diverse function in different cell types. While in naïve T cells it might help in minimizing the metabolic activity by controlling intracellular NAD+ levels, in activated effector T cells it may possibly regulate cellular proliferation and cytokine production by affecting T cell receptor signaling and/or co-stimulatory signaling. During immune cell activation, CD38 is often found to be colocalized with the prime signaling receptors in the immune synapse during immune cell activation in all immune cell types (i.e. TCRs in T cells, CD19 in B cells, CD16 in NK cells and CD81 in Monocytes) (7, 38). In T cells, CD38 associates with Lck through the cytoplasmic tail and the Src homology 2 domain and transduces the signal for T cell activation (39). Also, it is well-established that CD38, utilizes intracellular NAD+ to generate second messenger cADPR, which in turn helps in Ca2+ signaling required for T cell activation (40). In this study, we reported that upon antigen exposure cytokine producing cells of PWH expressed higher CD38 compared to its global expression on T cells and that inhibiting CD38 reduces cytokine production by these cells indicating a different role of CD38 in the cytokine producing cells. It would be interesting to verify the impact of CD38 inhibition on Ca2+ signaling in the antigen exposed effector cells, but we could not delineate that due to shortage of cells number. Nonetheless, such proinflammatory cytokine response by high CD38 expressing effector cells is likely to result in an enhanced immune activation state, that could lead to HIV associated end organ disease. However, the findings raise the question of how these activated T cells maintain their intracellular NAD+ levels in the presence of high CD38 expression. Previously, it was reported that after mitogenic T cell activation, various key enzymes in the NAD+ synthesis pathway e.g., NMN adenylyltransferase (NMNAT) and nicotinamide phosphoribosyl transferase (Nampt)), are upregulated in T lymphocytes, increasing NAD+ levels in these cells (41, 42). Thus, we speculate that CD38 mediated NAD+ reduction in activated T cells is replenished by *de novo* NAD+ synthesis or NAD+ salvage pathway, maintaining these effector cells metabolically active. However, the mechanism of action of CD38 in B cells and NK cells during pathological conditions remain ambiguous.

The observation that the naïve T cell compartment exhibited higher CD38 expression compared to its effector counterparts might have significant clinical implications since CD38 monoclonal antibody is used to treat patients with multiple myeloma via antibody-dependent cellular cytotoxicity and is currently being investigated in clinical trials for chronic lymphocytic leukemia (8, 10). Recently, Krejcik et al. reported that multiple myeloma patients treated with daratumumab had significant decreases in naïve T cells (43), further supporting higher CD38 expression among naïve T cells. Loss of naïve T cells could be detrimental in adult individuals since it is a hallmark of immune aging and likely to be associated with increased susceptibility to chronic infections, autoimmune diseases, cardiovascular disease, and cancers (44–50).

In conclusion, CD38 exhibits functional diversity in T cells depending on the context of differentiation and activation status of the T lymphocytes. While, during physiological condition, higher CD38 expression on naïve T cells modulates their metabolic activity and mitochondrial function to keep them in a quiescent state, in effector T cells, it is required for signal transduction to execute effector function upon antigenic stimulation. During pathological conditions such as HIV, CD38 is overexpressed maintaining a sustained immune activation state with proinflammatory cytokine secretion. Despite optimal control of HIV replication *in vivo*, PWH still suffer from significant comorbidities such as end organ diseases resulting from immune inflammation responses. This study provides a scientific rationale to the mechanisms involving altered CD38-NAD+-mitochondrial axis that could serve as a potential therapeutic target to reduce inflammation/activation induced damage to end organ disease and even accelerated gaining observed in PWH.

## Data availability statement

The original contributions presented in the study are included in the article/**Supplementary Material**. Further inquiries can be directed to the corresponding author.

## Ethics statement

The studies involving human participants were reviewed and approved by University of Maryland, Baltimore Institutional Review Board. The patients/participants provided their written informed consent to participate in this study.

## Author contributions

AG designed the methods, performed the experiments, analyzed data, interpreted the results, and wrote the manuscript. AK and KR performed experiments and critically edited the manuscript. PM provided clinical expertise and HIV samples and edited the manuscript. AS edited and formatted the manuscript. BP and SK participated in study design, critically edited the manuscript, provided resources and funding. All authors contributed to the article and approved the submitted version.

## Supplementary material

Supplementary Figure 1 Comparison of the ratio of intracellular NAD+/NADH level. The ratio of intracellular NAD+/NADH level were compared between naïve and effector CD3+ T cell subsets. Paired t-test (for normal distribution, parametric) was performed for statistical analysis. ns – not significant.

Supplementary Figure 2 Assessment of Mitotracker Green (MTG) and Mitosox Red (MSR) co-expression among the T cell subsets of HDs. **(A)** Gating strategy for MTG and MSR staining among naïve and effector subsets of CD4+ T cells and CD8+ T cells. Comparison of MSR-MFI on high MTG (MTG-hi) and low MTG (MTG-lo) expressing naïve and effector subsets of CD4+ **(B)** and CD8+ **(C)** T cells. In each plot, dots represent individual samples. Paired t-test (ratio) was performed for statistical analysis. P values; * <0.05, ** <0.01, ns – not significant.

Supplementary Figure 3 Frequencies of CD38+ cells among Naïve and Effector subsets of **(A)** CD4+ and **(B)** CD8+ T cells in PBMC of HDs and PWH. Cells were gated following the same gating strategy as in . Mann-Whitney test was performed for statistical analysis. ns – not significant.

## References

[1] Reinherz EL Kung PC Goldstein G Levey RH Schlossman SF. Discrete stages of human intrathymic differentiation: analysis of normal thymocytes and leukemic lymphoblasts of T-cell lineage. Proc Natl Acad Sci U.S.A. (1980) 77(3):1588–92. doi: 10.1073/pnas.77.3.1588 PMC348542 6966400

[2] Shubinsky G Schlesinger M. The CD38 lymphocyte differentiation marker: new insight into its ectoenzymatic activity and its role as a signal transducer. Immunity (1997) 7(3):315–24. doi: 10.1016/S1074-7613(00)80353-2 9324352

[3] Sandoval-Montes C Santos-Argumedo L. CD38 is expressed selectively during the activation of a subset of mature T cells with reduced proliferation but improved potential to produce cytokines. J Leukoc Biol (2005) 77(4):513–21. doi: 10.1189/jlb.0404262 15618297

[4] Frasca L Fedele G Deaglio S Capuano C Palazzo R Vaisitti T. CD38 orchestrates migration, survival, and Th1 immune response of human mature dendritic cells. Blood (2006) 107(6):2392–9. doi: 10.1182/blood-2005-07-2913 16293598

[5] Choe CU Lardong K Gelderblom M Ludewig P Leypoldt F Koch-Nolte F. CD38 exacerbates focal cytokine production, postischemic inflammation and brain injury after focal cerebral ischemia. PloS One (2011) 6(5):e19046. doi: 10.1371/journal.pone.0019046 21625615 PMC3097994

[6] Garcia-Rodriguez S Rosal-Vela A Botta D Cumba Garcia LM Zumaquero E Prados-Maniviesa V. CD38 promotes pristane-induced chronic inflammation and increases susceptibility to experimental lupus by an apoptosis-driven and TRPM2-dependent mechanism. Sci Rep (2018) 8(1):3357. doi: 10.1038/s41598-018-21337-6 29463868 PMC5820326

[7] Malavasi F Deaglio S Funaro A Ferrero E Horenstein AL Ortolan E. Evolution and function of the ADP ribosyl cyclase/CD38 gene family in physiology and pathology. Physiol Rev (2008) 88(3):841–86. doi: 10.1152/physrev.00035.2007 18626062

[8] Ejaz K Roback JD Stowell SR Sullivan HC. Daratumumab: beyond multiple myeloma. Transfus Med Rev (2021) 35(3):36–43. doi: 10.1016/j.tmrv.2021.06.002 34312046

[9] Nooka AK Kaufman JL Hofmeister CC Joseph NS Heffner TL Gupta VA. Daratumumab in multiple myeloma. Cancer (2019) 125(14):2364–82. doi: 10.1002/cncr.32065 30951198

[10] van de Donk N. Immunomodulatory effects of CD38-targeting antibodies. Immunol Lett (2018) 199:16–22. doi: 10.1016/j.imlet.2018.04.005 29702148

[11] Dhillon S. Isatuximab: first approval. Drugs (2020) 80(9):905–12. doi: 10.1007/s40265-020-01311-1 32347476

[12] Hong A Eduafo A Schmikla H Brown G Ravi G de Lima M. Analyzing risk of infection with anti-CD38 monoclonal antibody therapy for patients with multiple myeloma. Blood (2020) 136(Supplement 1):49–9. doi: 10.1182/blood-2020-140544

[13] Tarrago MG Chini CCS Kanamori KS Warner GM Caride A de Oliveira GC. A potent and specific CD38 inhibitor ameliorates age-related metabolic dysfunction by reversing tissue NAD(+) decline. Cell Metab (2018) 27(5):1081–1095.e10. doi: 10.1016/j.cmet.2018.03.016 29719225 PMC5935140

[14] Camacho-Pereira J Tarrago MG Chini CCS Nin V Escande C Warner GM. CD38 dictates age-related NAD decline and mitochondrial dysfunction through an SIRT3-dependent mechanism. Cell Metab (2016) 23(6):1127–39. doi: 10.1016/j.cmet.2016.05.006 PMC4911708 27304511

[15] Covarrubias AJ Kale A Perrone R Lopez-Dominguez JA Pisco AO Kasler HG. Senescent cells promote tissue NAD+ decline during ageing via the activation of CD38+ macrophages. Nat Metab (2020) 2(11):1265–83. doi: 10.1038/s42255-020-00305-3 PMC7908681 33199924

[16] Chou JP Ramirez CM Wu JE Effros RB. Accelerated aging in HIV/AIDS: novel biomarkers of senescent human CD8+ T cells. PloS One (2013) 8(5):e64702. doi: 10.1371/journal.pone.0064702 23717651 PMC3661524

[17] Gindin Y Gaggar A Lok AS Janssen HLA Ferrari C Subramanian GM. DNA Methylation and immune cell markers demonstrate evidence of accelerated aging in patients with chronic hepatitis b virus or hepatitis c virus, with or without human immunodeficienct virus Co-infection. Clin Infect Dis (2021) 73(1):e184–90. doi: 10.1093/cid/ciaa1371 PMC8427715 32915202

[18] Giorgi JV Liu Z Hultin LE Cumberland WG Hennessey K Detels R. Elevated levels of CD38+ CD8+ T cells in HIV infection add to the prognostic value of low CD4+ T cell levels: results of 6 years of follow-up. the Los Angeles center, multicenter AIDS cohort study. J Acquir Immune Defic Syndr (1988) (1993) 6(8):904–12. 7686224

[19] Mathur P Kottilil S Pallikkuth S Frasca D Ghosh A. Persistent CD38 expression on CD8 + T lymphocytes contributes to altered mitochondrial function and chronic inflammation in people with HIV, despite ART. J Acquir Immune Defic Syndr (2022) 91(4):410–8. doi: 10.1097/QAI.0000000000003080 PMC9613598 36000933

[20] Najafi Fard S Schietroma I Corano Scheri G Giustini N Serafino S Cavallari EN. Direct-acting antiviral therapy enhances total CD4+ and CD8+ T-cells responses, but does not alter T-cells activation among HCV mono-infected, and HCV/HIV-1 co-infected patients. Clin Res Hepatol Gastroenterol (2018) 42(4):319–29. doi: 10.1016/j.clinre.2017.11.006 29279268

[21] Gonzalez VD Falconer K Blom KG Reichard O Morn B Laursen AL. High levels of chronic immune activation in the T-cell compartments of patients coinfected with hepatitis c virus and human immunodeficiency virus type 1 and on highly active antiretroviral therapy are reverted by alpha interferon and ribavirin treatment. J Virol (2009) 83(21):11407–11. doi: 10.1128/JVI.01211-09 PMC2772767 19710147

[22] Sherman GG Scott LE Galpin JS Kuhn L Tiemessen CT Simmank K. CD38 expression on CD8(+) T cells as a prognostic marker in vertically HIV-infected pediatric patients. Pediatr Res (2002) 51(6):740–5. doi: 10.1203/00006450-200206000-00013 12032270

[23] Benito JM Lopez M Lozano S Martinez P Gonzalez-Lahoz J Soriano V. CD38 expression on CD8 T lymphocytes as a marker of residual virus replication in chronically HIV-infected patients receiving antiretroviral therapy. AIDS Res Hum Retroviruses (2004) 20(2):227–33. doi: 10.1089/088922204773004950 15018711

[24] Dianzani U Funaro A DiFranco D Garbarino G Bragardo M Redoglia V. Interaction between endothelium and CD4+CD45RA+ lymphocytes. role of the human CD38 molecule. J Immunol (1994) 153(3):952–9. 7913116

[25] Covarrubias AJ Perrone R Grozio A Verdin E. NAD(+) metabolism and its roles in cellular processes during ageing. Nat Rev Mol Cell Biol (2021) 22(2):119–41. doi: 10.1038/s41580-020-00313-x PMC7963035 33353981

[26] Amjad S Nisar S Bhat AA Shah AR Frenneaux MP Fakhro K. Role of NAD(+) in regulating cellular and metabolic signaling pathways. Mol Metab (2021) 49:101195. doi: 10.1016/j.molmet.2021.101195 33609766 PMC7973386

[27] Lischke T Heesch K Schumacher V Schneider M Haag F Koch-Nolte F. CD38 controls the innate immune response against listeria monocytogenes. Infect Immun (2013) 81(11):4091–9. doi: 10.1128/IAI.00340-13 PMC3811837 23980105

[28] Partida-Sánchez S Cockayne DA Monard S Jacobson EL Oppenheimer N Garvy B. Cyclic ADP-ribose production by CD38 regulates intracellular calcium release, extracellular calcium influx and chemotaxis in neutrophils and is required for bacterial clearance in vivo. Nat Med (2001) 7(11):1209–16. doi: 10.1038/nm1101-1209 11689885

[29] Partida-Sánchez S Randall TD Lund FE. Innate immunity is regulated by CD38, an ecto-enzyme with ADP-ribosyl cyclase activity. Microbes Infect (2003) 5(1):49–58. doi: 10.1016/S1286-4579(02)00055-2 12593973

[30] Viegas MS do Carmo A Silva T Seco F Serra V Lacerda M. CD38 plays a role in effective containment of mycobacteria within granulomata and polarization of Th1 immune responses against mycobacterium avium. Microbes Infect (2007) 9(7):847–54. doi: 10.1016/j.micinf.2007.03.003 17533152

[31] Castellanos E Lanning NJ. Phosphorylation of OXPHOS machinery subunits: functional implications in cell biology and disease. Yale J Biol Med (2019) 92(3):523–31. PMC6747953 31543713

[32] Grayson JM Laniewski NG Lanier JG Ahmed R. Mitochondrial potential and reactive oxygen intermediates in antigen-specific CD8+ T cells during viral infection. J Immunol (2003) 170(9):4745–51. doi: 10.4049/jimmunol.170.9.4745 12707355

[33] Marek N Mysliwska J Raczynska K Trzonkowski P. Membrane potential of CD4+ T cells is a subset specific feature that depends on the direct cell-to-cell contacts with monocytes. Hum Immunol (2010) 71(7):666–75. doi: 10.1016/j.humimm.2010.05.002 20457202

[34] Jameson SC Masopust D. Understanding subset diversity in T cell memory. Immunity (2018) 48(2):214–26. doi: 10.1016/j.immuni.2018.02.010 PMC5863745 29466754

[35] Appay V Dunbar PR Callan M Klenerman P Gillespie GM Papagno L. Memory CD8+ T cells vary in differentiation phenotype in different persistent virus infections. Nat Med (2002) 8(4):379–85. doi: 10.1038/nm0402-379 11927944

[36] Zaunders JJ Munier ML Kaufmann DE Ip S Grey P Smith D. Early proliferation of CCR5(+) CD38(+++) antigen-specific CD4(+) Th1 effector cells during primary HIV-1 infection. Blood (2005) 106(5):1660–7. doi: 10.1182/blood-2005-01-0206 15905189

[37] Chun TW Justement JS Sanford C Hallahan CW Planta MA Loutfy M. Relationship between the frequency of HIV-specific CD8+ T cells and the level of CD38+CD8+ T cells in untreated HIV-infected individuals. Proc Natl Acad Sci U.S.A. (2004) 101(8):2464–9. doi: 10.1073/pnas.0307328101 PMC356973 14983032

[38] Munoz P Mittelbrunn M de la Fuente H Perez-Martinez M Garcia-Perez A Ariza-Veguillas A. Antigen-induced clustering of surface CD38 and recruitment of intracellular CD38 to the immunologic synapse. Blood (2008) 111(7):3653–64. doi: 10.1182/blood-2007-07-101600 18212246

[39] Cho YS Han MK Choi YB Yun Y Shin J Kim UH. Direct interaction of the CD38 cytoplasmic tail and the lck SH2 domain. Cd38 transduces T cell activation signals through associated lck. J Biol Chem (2000) 275(3):1685–90. doi: 10.1074/jbc.275.3.1685 10636863

[40] Kar A Mehrotra S Chatterjee S. CD38: T cell immuno-metabolic modulator. Cells (2020) 9(7). doi: 10.3390/cells9071716 PMC7408359 32709019

[41] Berger SJ Manory I Sudar DC Krothapalli D Berger NA. Pyridine nucleotide analog interference with metabolic processes in mitogen-stimulated human T lymphocytes. Exp Cell Res (1987) 173(2):379–87. doi: 10.1016/0014-4827(87)90278-3 2961586

[42] Bruzzone S Fruscione F Morando S Ferrando T Poggi A Garuti A. Catastrophic NAD+ depletion in activated T lymphocytes through nampt inhibition reduces demyelination and disability in EAE. PloS One (2009) 4(11):e7897. doi: 10.1371/journal.pone.0007897 19936064 PMC2774509

[43] Krejcik J Casneuf T Nijhof IS Verbist B Bald J Plesner T. Daratumumab depletes CD38+ immune regulatory cells, promotes T-cell expansion, and skews T-cell repertoire in multiple myeloma. Blood (2016) 128(3):384–94. doi: 10.1182/blood-2015-12-687749 PMC4957162 27222480

[44] Li M Yao D Zeng X Kasakovski D Zhang Y Chen S. Age related human T cell subset evolution and senescence. Immun Ageing (2019) 16:24. doi: 10.1186/s12979-019-0165-8 31528179 PMC6739976

[45] Saule P Trauet J Dutriez V Lekeux V Dessaint JP Labalette M. Accumulation of memory T cells from childhood to old age: central and effector memory cells in CD4(+) versus effector memory and terminally differentiated memory cells in CD8(+) compartment. Mech Ageing Dev (2006) 127(3):274–81. doi: 10.1016/j.mad.2005.11.001 16352331

[46] Weng NP Akbar AN Goronzy J. CD28(-) T cells: their role in the age-associated decline of immune function. Trends Immunol (2009) 30(7):306–12. doi: 10.1016/j.it.2009.03.013 PMC2801888 19540809

[47] Moro-Garcia MA Echeverria A Galan-Artimez MC Suarez-Garcia FM Solano-Jaurrieta JJ Avanzas-Fernandez P. Immunosenescence and inflammation characterize chronic heart failure patients with more advanced disease. Int J Cardiol (2014) 174(3):590–9. doi: 10.1016/j.ijcard.2014.04.128 24801091

[48] Xu L Yao D Tan J He Z Yu Z Chen J. Memory T cells skew toward terminal differentiation in the CD8+ T cell population in patients with acute myeloid leukemia. J Hematol Oncol (2018) 11(1):93. doi: 10.1186/s13045-018-0636-y 29986734 PMC6038290

[49] Yao D Xu L Tan J Zhang Y Lu S Li M. Re-balance of memory T cell subsets in peripheral blood from patients with CML after TKI treatment. Oncotarget (2017) 8(47):81852–9. doi: 10.18632/oncotarget.20965 PMC5669853 29137227

[50] Fang P Li X Dai J Cole L Camacho JA Zhang Y. Immune cell subset differentiation and tissue inflammation. J Hematol Oncol (2018) 11(1):97. doi: 10.1186/s13045-018-0637-x 30064449 PMC6069866

