# Supplementary figures and images for: CD38: an ecto-enzyme with functional diversity in T cells
Source: Front Immunol. 2023 Apr 27;14:1146791. doi: 10.3389/fimmu.2023.1146791 (PMC10172466; doi:10.3389/fimmu.2023.1146791)

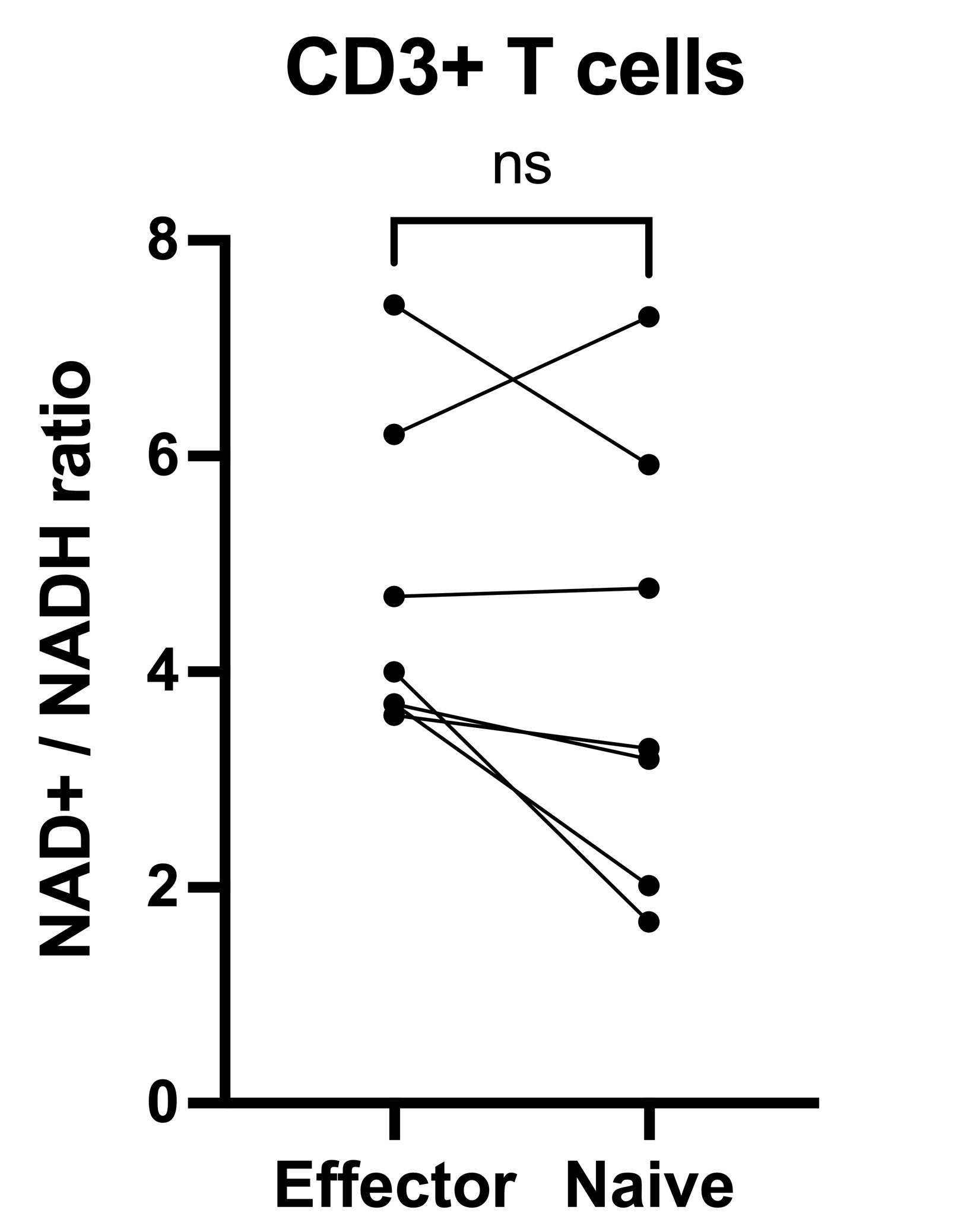

Supplement: Supplementary Figure 1 — Comparison of the ratio of intracellular NAD+/NADH level. The ratio of intracellular NAD+/NADH level were compared between naïve and effector CD3+ T cell subsets. Paired t-test (for normal distribution, parametric) was performed for statistical analysis. ns – not significant. [file Image_1.jpeg]

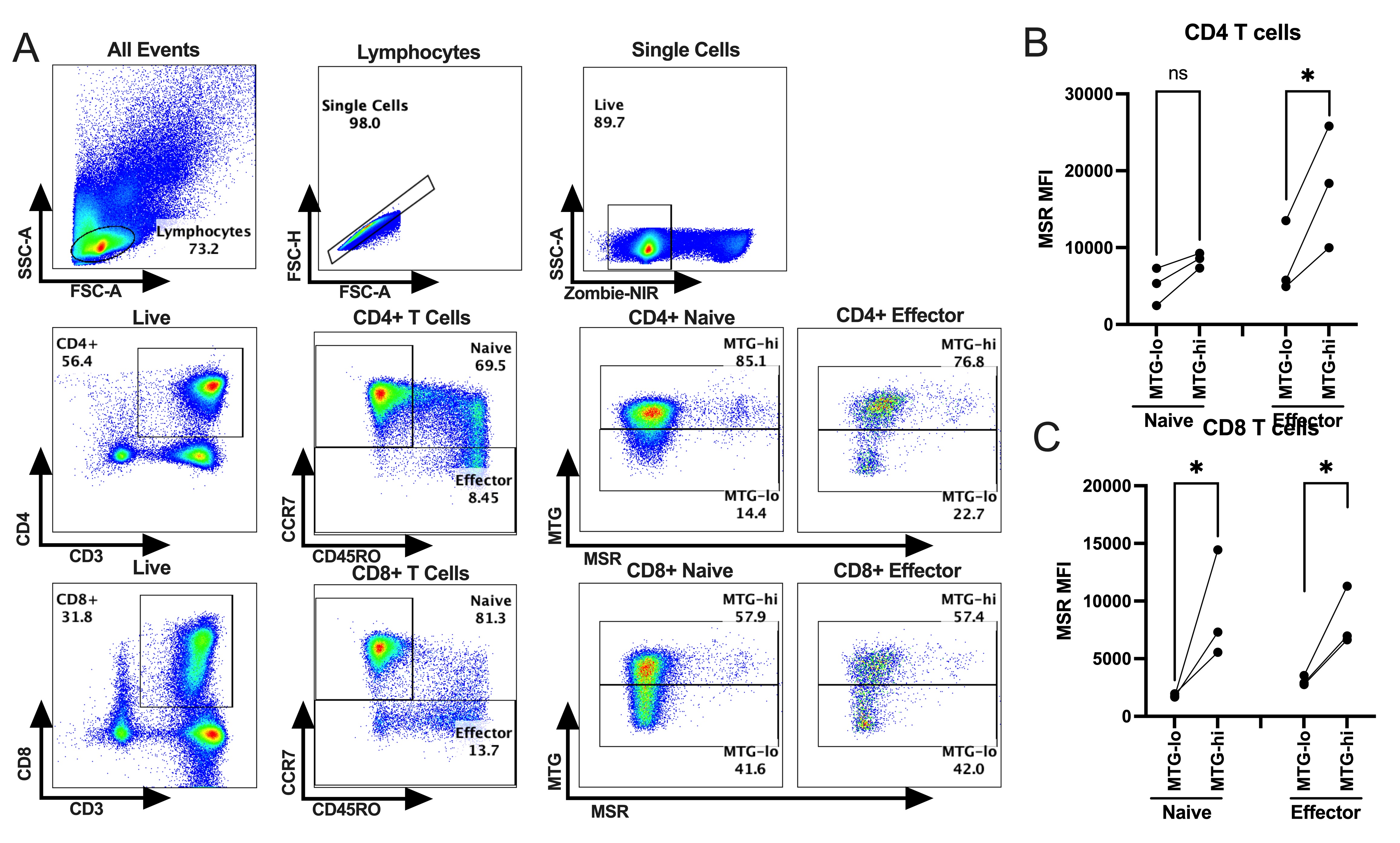

Supplement: Supplementary Figure 2 — Assessment of Mitotracker Green (MTG) and Mitosox Red (MSR) co-expression among the T cell subsets of HDs. (A) Gating strategy for MTG and MSR staining among naïve and effector subsets of CD4+ T cells and CD8+ T cells. Comparison of MSR-MFI on high MTG (MTG-hi) and low MTG (MTG-lo) expressing naïve and effector subsets of CD4+ (B) and CD8+ (C) T cells. In each plot, dots represent individual samples. Paired t-test (ratio) was performed for statistical analysis. P values; * <0.05, ** <0.01, ns – not significant. [file Image_2.jpeg]

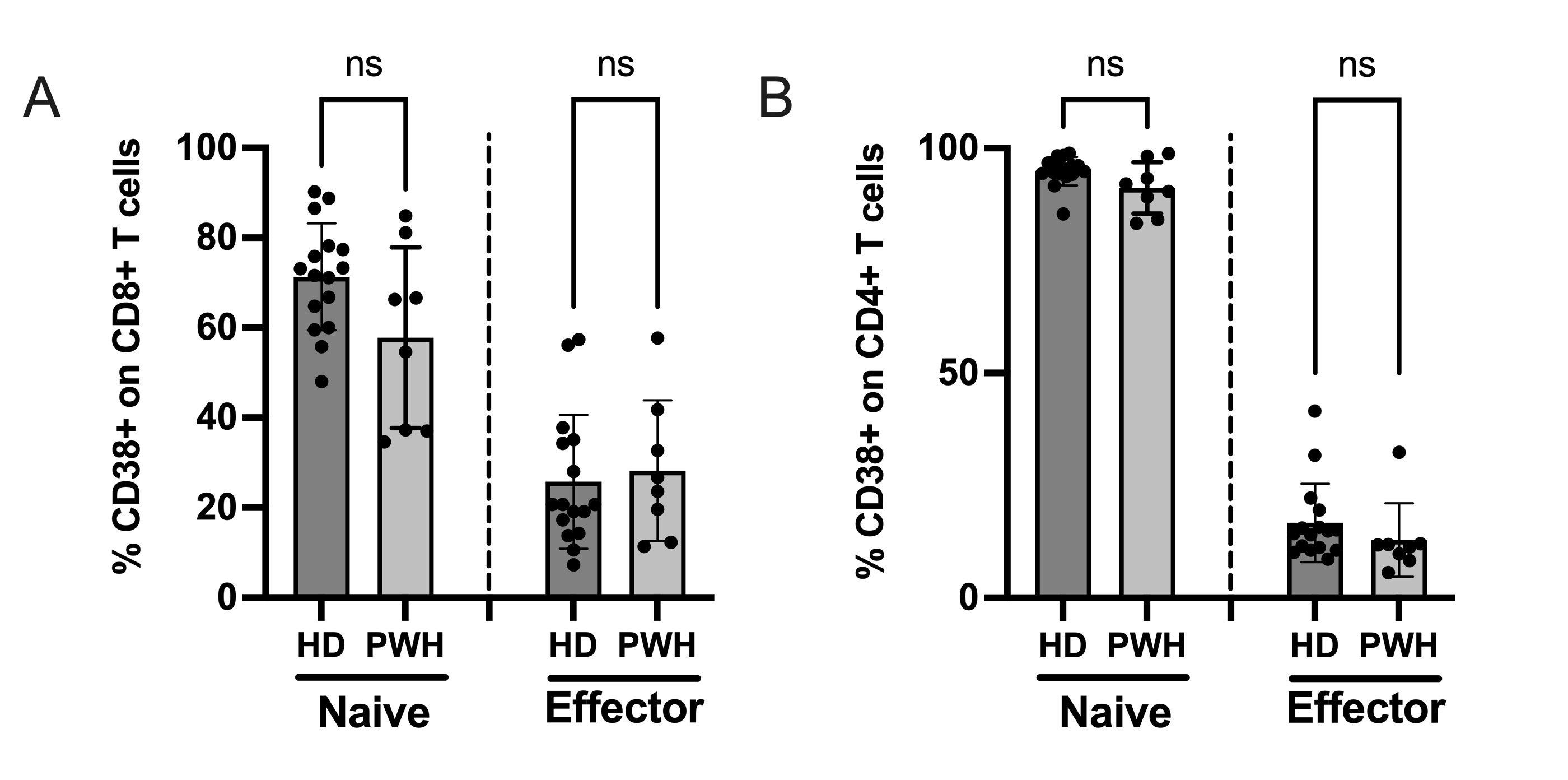

Supplement: Supplementary Figure 3 — Frequencies of CD38+ cells among Naïve and Effector subsets of (A) CD4+ and (B) CD8+ T cells in PBMC of HDs and PWH. Cells were gated following the same gating strategy as in . Mann-Whitney test was performed for statistical analysis. ns – not significant. [file Image_3.jpeg]
